# Identification and characterization of a spontaneous ovarian carcinoma in Lewis rats

**DOI:** 10.1186/1757-2215-3-9

**Published:** 2010-03-31

**Authors:** Allison C Sharrow, Brigitte M Ronnett, Christopher J Thoburn, James P Barber, Robert L Giuntoli, Deborah K Armstrong, Richard J Jones, Allan D Hess

**Affiliations:** 1 The Sidney Kimmel Comprehensive Cancer Center, Johns Hopkins University School of Medicine, Baltimore, MD, USA; 2 Department of Pathology, Johns Hopkins University School of Medicine, Baltimore, MD, USA; 3 Department of Gynecology and Obstetrics, Johns Hopkins University School of Medicine, Baltimore, MD, USA

## Abstract

**Background:**

Ovarian carcinoma is the fourth most common cause of death from cancer in women. Limited progress has been made toward improving the survival rate of patients with this disease in part because of the lack of a good animal model. We present here a model of spontaneous ovarian carcinoma arising in a normal Lewis rat.

**Methods:**

A spontaneously occurring tumor of the left ovary was found in a normal Lewis rat during necropsy, which was sectioned for histological examination and placed into single cell suspension. Tumor cells were passaged *in vivo *by intraperitoneal injection into immunocompetent Lewis rats, and *in vitro *culture resulted in generation of a cell line. Tumor cells were examined by flow cytometry for expression of estrogen receptor α, progesterone receptor, androgen receptor, her-2/neu, epithelial cell adhesion molecule, and CA125. β-catenin expression and cellular localization was assessed by immunocytochemistry. RNA was harvested for gene expression profiling and studying the expression of cytokines.

**Results:**

The tumor, designated FNAR, could be serially transplanted into Lewis rats and propagated as a cell line *in vitro*, maintaining the properties of the original tumor. The FNAR cells displayed striking morphologic similarities to human ovarian carcinoma, resembling the endometrioid carcinoma subtype of surface epithelial neoplasms. The cells expressed estrogen receptor α, progesterone receptor, androgen receptor, her-2/neu, epithelial cell adhesion molecule, CA125, and nuclear β-catenin. A gene expression profile showed upregulation of a number of genes that are also upregulated in human ovarian carcinoma.

**Conclusion:**

This reliable model of ovarian carcinoma should be helpful in better understanding the biology of the disease as well as the development of novel treatment strategies.

## Background

Ovarian cancer is the fifth most commonly diagnosed cancer in women and the fourth most common cause of death from cancer [1]. The high mortality can be attributed to the high percentage of affected women presenting at an advanced stage, with spread within the peritoneal cavity [2,3]. With current therapies, including surgical debulking and platinum-based chemotherapy, patients in stage III or stage IV only have a 20% chance of long-term survival [2,3]. Better understanding ovarian carcinoma biology, as well as the development of new therapies for the disease, has been hampered by the lack of suitable animal models.

Current ovarian cancer models fall into three broad categories: rare spontaneous carcinomas, induced tumors, and human xenografts [4]. Although these models have allowed researchers to gain valuable insights into the biology of ovarian cancer, each model exhibits important limitations [4,5]. Spontaneous ovarian cancer has been observed in mice, rats, and hens [6-8]. The drawback to these models is that the cancers tend to occur at an advanced age and at similar low frequencies as in humans. The low incidence and the length of time required for the development of these tumors render them of limited use for studying the biology and treatment of ovarian carcinoma. Induced tumor models circumvent these problems but create their own artificial systems, which may not accurately reflect the human disease. In one model of *in vitro *transformation, ovarian surface epithelium cells are subcloned until they exhibit the loss of contact inhibition, the capacity for substrate-independent growth, cytogenetic abnormalities, and the ability to form tumors when injected subcutaneously and/or intraperitoneally into athymic mice [9]. This model, though, fails to account for critical interactions between the cancer cells and the host. Also, it is uncertain if these cells or their malignant transformation are representative of normal human cells or clinical disease.

Animal models have been generated by expressing simian virus 40 large T antigen [10], by inactivating p53 and Rb1 [11], by inactivating p53 and activating an oncogene [12], and through hormone treatment [13-15]. The high rate of cancer development in these animals makes these models attractive, but they may not reliably represent human cancer because a majority of these genetic changes usually do not occur in patients. Xenografts of human cancers have undergone continuous improvement over the past twenty years [16-19]. These models allow for direct examination of the human cancer but do not allow the study of the early stages of the cancer. These models also rely on an immune-deficient host, which eliminates the interaction between the cancer and the immune system.

We present a new model of ovarian carcinoma, designated FNAR, that spontaneously developed in an untreated, previously normal Lewis rat. The tumor could be serially passaged both *in vivo *as malignant ascites in rats and *in vitro*. Importantly, the biologic characteristics of the tumor closely paralleled one type of human ovarian carcinoma.

## Methods

### Animals

Female Lewis strain rats aged 4-6 weeks (purchased from Charles River Breeding Laboratories, Inc., Wilmington, MA) were kept in sterile micro-isolator cages and fed food and water *ad libitum*. The institutional guidelines of Johns Hopkins University concerning the care and use of research animals were followed. The animals were challenged intraperitoneally with graded numbers of FNAR cells and monitored daily for abdominal swelling. At various intervals after tumor challenge or when animals appeared moribund (pallor, lethargy, and marked abdominal distension), the animals were sacrificed by CO_2 _asphyxiation and the cells within the peritoneal cavity harvested by flushing the abdomen with 35 milliliters of sterile phosphate buffered saline (PBS, Grand Island Biological Co., Gibco BRL, Grand Island, NY). At sacrifice, the animals were examined for tumor growth and tissues taken for histological examination.

### *In vitro *propagation and growth curve

A cell line (FNAR) that grows *in vitro *as an adherent monolayer was established by culture in RPMI 1640 (Gibco) supplemented with 10% fetal calf serum in 30 ml tissue culture flasks (Corning Flask 3056, Corning Inc., Corning NY). Cells used for experiments were low passage and maintained in culture for one to three months. The doubling time of the cell line was measured by plating 10^4 ^cells into macrotiter wells then harvesting and counting at 19.5, 43.5, and 115.5 hours.

### Flow Cytometric Analysis

Flow cytometry was utilized to assess *in vitro *FNAR cells for expression of known phenotypic markers. Briefly, 5 × 10^5 ^tumor cells were incubated in polystyrene tubes. Analysis of the intracellular antigens estrogen receptor α, progesterone receptor, and androgen receptor first required fixation in 2% formaldehyde (Polysciences, Warrington, PA) in phosphate buffered saline (PBS, Gibco Invitrogen, Carlsbad, CA) for 15 minutes at 4°C followed by permeabilization with 0.1% Triton-X-100 (Sigma-Aldrich, St. Louis, MO) in PBS for 15 minutes at 4°C. The cells were then incubated for 30 minutes at 4°C with commercially purchased murine monoclonal antibodies. The concentrations of antibodies used are as follows: estrogen receptor (ER) α at 8 μg/10^6 ^cells (Abcam, Cambridge, MA), progesterone receptor (PR) at 16 μg/10^6 ^cells (Affinity Bioreagents, Golden, CO), or androgen receptor (AR) at 2 μg/10^6 ^cells (Pharmingen, San Diego, CA). The cells were washed and counterstained with phycoerythrin (PE) rat anti-mouse IgG_1 _(Becton Dickinson, San Jose, CA) at 125 ng/10^6 ^cells for 30 minutes at 4°C. Commercially purchased murine monoclonal antibody to the rat c-neu oncogene product (Calbiochem, San Diego, CA) was used at 1 μg/10^6 ^cells and was counterstained with PE rat anti-mouse IgG_2a+b _(Becton Dickinson, San Jose, CA) at 30 ng/10^6 ^cells for 30 minutes at 4°C. Tumor cells incubated with secondary antibody alone served as a negative control. Epithelial cell adhesion molecule (EPCAM) expression was analyzed using a PE-conjugated antibody (Santa Cruz, Santa Cruz, CA) at 1 μg/10^6 ^cells with mouse IgG_1_-PE as a negative control (Becton Dickinson, San Jose, CA). A commercially available rabbit polyclonal antibody to CA125 (Abbiotec, San Diego, CA) was used at 2 μg/10^6 ^cells and counterstained with 1 μg/10^6 ^cells APC goat anti-rabbit IgG (Invitrogen Molecular Probes, Carlsbad, CA). The cells were analyzed on a Becton-Dickinson FACSCalibur flow cytometer and data was analyzed using FlowJo (Tree Star, Inc, Ashland, OR).

### Immunocytochemistry

FNAR cells were plated onto four-well CultureSlides (BD Falcon, San Jose, CA). Cells were fixed in 2% formaldehyde in PBS for 20 minutes followed by permeabilization in 0.5% Triton X-100 in PBS for 10 minutes. Cells were then incubated with a mouse monoclonal antibody to beta-catenin conjugated to Cy3 (Abcam, Cambridge, MA) at 6 μg/ml for one hour and counterstained with 500 ng/ml DAPI for five minutes (Invitrogen Molecular Probes, Carlsbad, CA). Images were captured using the Nikon Eclipse E800 (Tokyo, Japan) at 200× magnification with standard filters for DAPI and Cy3, the DS-QiMc digital camera (Nikon, Tokyo, Japan), and the Advanced Research Elements AR 3.0 software (Nikon, Tokyo, Japan).

### Gene Expression Analysis by cDNA Microarrays

RNA was extracted and purified from cell lysates of 1-5 × 10^5 ^*in vitro *FNAR tumor cells and the REH cell line of normal rat endothelial cells, as a control, with 500 μl Trizol reagent (Invitrogen, Carlsbad, CA). Tissue samples were frozen in liquid nitrogen and pulverized with a mortar and pestle. The powder was dissolved in Trizol and centrifuged. Purified RNA was dissolved in 20 μl diethyl-pyrocarbonate-treated distilled water. The resulting RNA was analyzed at the Johns Hopkins microarray core. RNA from control and experimental samples was processed using the RNA amplification protocol described by Affymetrix (Affymetrix Expression Manual). Briefly, 5 μg of total RNA was used to synthesize first strand cDNA using the SuperScript Choice System (Invitrogen, Carlsbad, California) and oligonucleotide primers with 24 oligo-dT plus the T7 promoter (Proligo LLC, Boulder, Colorado). Following the double stranded cDNA synthesis, the product was purified by phenol-chloroform extraction and biotinilated anti-sense cRNA was generated through *in vitro *transcription using the BioArray RNA High Yield Transcript Labeling kit (ENZO Life Sciences Inc., Farmingdale, New York). Fifteen μg of the biotinilated cRNA was fragmented at 94°C for 35 minutes in buffer (100 mM Tris-acetate, pH 8.2, 500 mM potassium acetate, and 150 mM magnesium acetate), and 10 μg of total fragmented cRNA was hybridized to the Affymetrix GeneChip rat 230 2.0 array (Santa Clara, CA) for 16 hours at 45°C with constant rotation (60 rpm). Affymetrix Fluidics Station 450 was then used to wash and stain the chips with a streptavidin-phycoerythrin conjugate. The staining was then amplified as follows: blocking was performed using goat IgG, then a biotinilated anti-streptavidin antibody (goat) was bound to the initial staining, and amplification was completed by the addition of a streptavidin-phycoerythrin conjugate. Fluorescence was detected using the Affymetrix 3000 7G GeneArray Scanner and image analysis of each GeneChip was done through the GeneChip Operating System 1.4.0 (GCOS) software from Affymetrix using the standard default settings. For comparison between different chips, global scaling was used to scale all probesets to a user defined target intensity (TGT) of 150.

### Quantitative RT-PCR for Cytokine Expression

Quantitative RT-PCR (Taqman, Applied Biosystems, ABI, Foster City, CA) was utilized to assess levels of cytokine mRNA transcripts of *in vitro *FNAR cells as previously described [20]. The oligonucleotide primers and fluoresceinated probes for the cytokine genes (IL-6, IL-12, and IL-18), ER, PR, and stathmin were purchased from ABI. Data were analyzed in real-time with Sequencer Detection version 1.6 software, with the results normalized against mRNA transcripts for the housekeeping gene glyceraldehyde-3-phosphate dehydrogenase (GADPH).

## Results

### Description of proband

**Figure 1:**
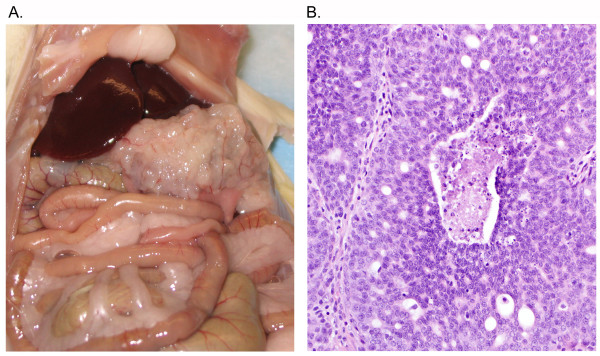
**Gross and histologic examination of proband**. Intraperitoneal tumor arising spontaneously in a Lewis rat has pathologic appearance of an ovarian adenocarcinoma. (A) Proband shows tumor of the left ovary and intraperitoneal tumor studding. (B) Histology reveals an adenocarcinoma.

Examination of a normal female Lewis rat sacrificed for harvesting normal splenic T cells showed a spontaneously occurring tumor (approximately 0.5 cm^3^) derived from the left ovary and attached to and invading the abdominal wall (Figure 1A). In addition, tumor studding was observed at several sites on the wall of the peritoneum, and ascites was present. Histologic evaluation revealed an epithelial neoplasm with features most consistent with an adenocarcinoma (Figure 1B). The tumor was composed of nests displaying admixed cribriform and solid architecture. The tumor cells had modest amounts of amphophilic/eosinophilic cytoplasm and relatively uniform, moderately atypical oval nuclei that were predominantly vesicular to modestly hyperchromatic with small nucleoli. Occasional mitotic figures and apoptotic bodies were noted, as was focal necrosis. Based on analogy to human ovarian epithelial tumors, this tumor most closely resembled a moderately differentiated endometrioid carcinoma (a cribriform variant of that subtype, with cells being less columnar than the classical human endometrioid carcinoma), with disease distribution paralleling a typical high-stage (human FIGO stage IIIB) ovarian carcinoma. Lymphocyte infiltration into the tumor mass was minimal at best, although numerous lymphocytes were present in the peritoneal fluid. The tumor was excised and pushed through a 100 micron wire mesh screen to obtain a single cell suspension.

### *In vivo *and *in vitro *growth characteristics

Normal Lewis rats were given either intraperitoneal (IP) or subcutaneous injection of graded numbers (5 × 10^4^, 1 × 10^5^, 5 × 10^5^, or 1 × 10^6^) of tumor cells. The animals were monitored daily for overall general health as well as degree of abdominal extension. The tumor repeatedly failed to grow subcutaneously, even with the administration of systemic immunosuppression (Cyclosporine, 10 mg/kg/d) or passage into thymectomized animals. However, all rats became moribund at 150-160 days after IP injection with 5 × 10^5 ^or 1 × 10^6 ^cells (Table 1). Rats injected with 1 × 10^5 ^cells became moribund around 175 days. Rats receiving IP injections of 5 × 10^4 ^cells generally did not appear ill by 6 months, but tumor cells were detected in the peritoneal cavity when sacrificed on day 175. Tumor growth recapitulated that seen in the initial rat with IP tumoral masses adhering to all of the visceral organs and the abdominal wall. Histologically, the tumors appeared to be of epithelial origin. Affected rats also showed enlargement of the ovaries and fallopian tubes, with a marked increase in vascularization. Successful serial passage was conducted by IP challenge with 1 × 10^5 ^tumor cells harvested by flushing of the peritoneal cavity.

**Table 1:** Survival after intraperitoneal injection of FNAR cells.

Survival Following Tumor Challenge		
No. of Cells Injected	No. of Animals	Survival - Days (No. of Animals)
5 × 10^4^	N = 6	175 (6)
1 × 10^5^	N = 8	150 (4) 155 (3), 160 (1)
5 × 10^5^	N = 6	155 (2), 160 (4)
1 × 10^6^	N = 6	150 (5), 152 (1)

The survival time of rats corresponds to the number of FNAR cells injected intraperitoneally. Animals were observed daily for general health and abdominal extension. The animals were sacrificed upon becoming moribund, which was characterized by extreme lethargy, paleness, and abdominal extension. The abdominal cavity was examined histologically for the presence of tumor cells in the peritoneal fluid and for tumor masses attached to the visceral organs and the abdominal wall.

**Figure 2:**
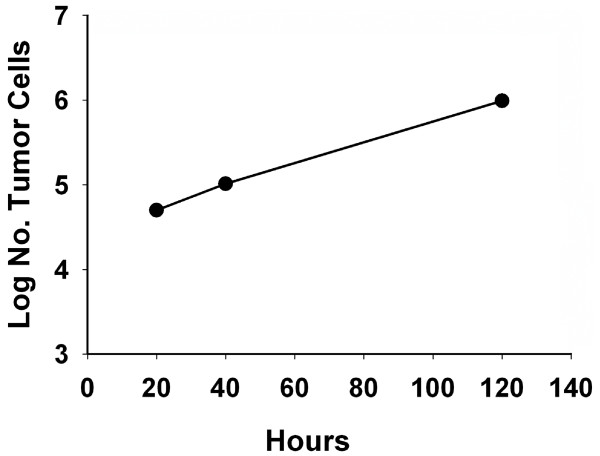
***In vitro *growth characteristics**. *In vitro *doubling time was measured by plating 10^4 ^cells into large flat bottom macrotiter wells. At the designated intervals, cells were harvested and counted. Data is presented as log number of tumor cells versus growth time. The slope of the line represents an estimate of the doubling time.

The doubling time of the FNAR cell line was measured by plating 10^4 ^cells into macrotiter wells then harvesting and counting at 19.5, 43.5, and 115.5 hours (Figure 2). The slope of the line of log number of tumor cells versus hours estimates a doubling time of 22.9 hours.

### Biological characterization of FNAR

**Figure 3:**
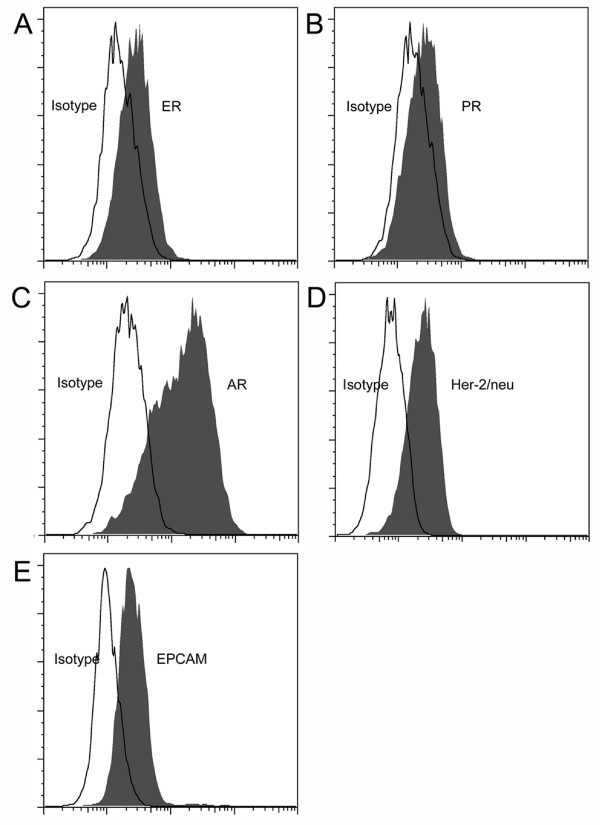
**FNAR expression of ER, PR, AR, Her-2/neu, and EPCAM**. Flow cytometric evaluation of FNAR cells for expression of (A) ER, (B) PR, (C) AR, (D) Her-2/neu, and (E) EPCAM. In all five graphs, isotypic control is shown with a solid line and the antibody of interest is shown with a shaded area.

**Figure 4:**
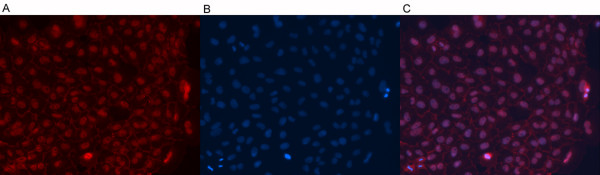
**FNAR expression of β-catenin**. FNAR cells were stained with (A) β-catenin and (B) DAPI. The third panel (C) shows an overlay of the two images.

ER is detected in 60-90% of ovarian carcinomas [21-25], 25-50% express PR [21,23-26], and 45% expressed both [23,25]. AR is expressed in 50-70% of ovarian carcinomas [24,26]. Accordingly, in the appropriate clinical and pathologic setting, sex hormone receptor expression is characteristic of ovarian carcinoma [25,27]. The tumor expressed ER, PR, and AR by flow cytometry (Figure 3A-C), with ER and PR confirmed by PCR (data not shown). The tumor also expressed her-2/neu (Figure 3D), which is expressed in 25-35% of ovarian carcinomas [28,29]. The epithelial origin of this carcinoma was confirmed by its expression of EPCAM (Figure 3E). Consistent with previous reports of endometrioid carcinoma, FNAR cells display cell-surface expression of CA125 (MUC16, data not shown) [30]. FNAR cells also show nuclear staining of β-catenin (Figure 4), which is strongly associated with the endometrioid subtype [31].

Gene expression profiling demonstrated that FNAR gene expression was similar to that reported for human ovarian carcinoma (Table 2). Metallothioneins are generally not found at immunohistochemically detectable levels in normal cells, but their expression increases in ovarian carcinoma with increasing grade [32-34]. Metallothionein I was overexpressed 11.38-fold in FNAR cells when compared to endothelial cells, and metallothionein II showed 3.56-fold increased expression. Thioredoxin expression correlates with cis-diaminedichloroplatinum resistance [35] and is expressed in FNAR cells 3.07-fold higher than in endothelial cells. Stathmin regulates microtubules during the formation of the mitotic spindle and is not expressed at detectable levels in normal cells; however, high-level expression is generally seen in ovarian carcinoma [36-38]. Accordingly, stathmin expression was 3.23-fold higher in FNAR cells than in endothelial cells. This data was confirmed by PCR (data not shown). A nuclear factor that it is involved in cell cycle progression, b-myb, is also highly expressed in both FNAR cells (3.33-fold) and human ovarian carcinoma [39].

**Table 2:** Gene chip analysis of FNAR.

Gene Expression Profiling of FNAR Cells		
Gene Description	EST Accession #	Relative Expression
Metallothionein I	AW141679	11.38
Metallothionein II	AW916991	3.56
Thioredoxin	AW140607	3.07
Stathmin	BF281472	3.23
b-myb	RGIAC37	3.33

Gene chip analysis of FNAR shows similarities to human ovarian carcinoma. RNA was harvested from FNAR and REH endothelial cell lines and analyzed by GeneChip at a Johns Hopkins core facility. Data are presented as the relative expression of the gene in FNAR compared to expression in endothelial cells.

**Figure 5:**
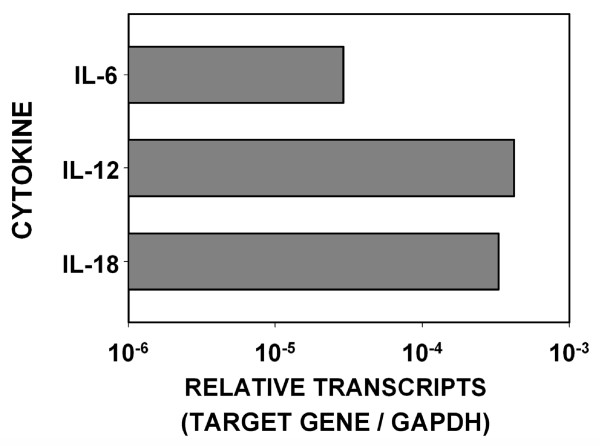
**FNAR expression of IL-6, IL-12, and IL-18**. FNAR tumor cells express IL-6, IL-12, and IL-18. Expression was assessed by qPCR. Data are standardized against GAPDH.

High levels of interleukin-6 (IL-6), a proinflammatory cytokine and hematopoietic growth factor, are found in both normal ovarian epithelium and human ovarian carcinoma [40,41]. Interleukin-18 (IL-18) is a proinflammatory cytokine that stimulates interferon-γ production. Ovarian carcinoma expresses IL-18, but it is predominantly the pro-IL-18 form [42]. Interleukin-12 (IL-12) is a cytokine that encourages a T_h_1 immune response. IL-12 has been detected in ascites fluid and serum of ovarian cancer patients [43], although no reports have examined the expression of IL-12 by the ovarian carcinoma cells themselves. Expression of all three cytokines by FNAR cells was detected by real time RT-PCR (Figure 5).

## Discussion

We present here a model of ovarian carcinoma, designated FNAR, that arose spontaneously in a normal Lewis rat. Importantly, FNAR's biology closely parallels the human disease. IP transplantation into rats produces malignant ascites and peritoneal carcinomatosis, leading to death at 5-6 months. The tumor only develops in the peritoneal cavity, suggesting the tumor microenvironment is intact during formation. Cells from the tumor can be easily passaged *in vitro*, and the cell line shows similar growth characteristics when returned to rats. Its morphology and expression of EPCAM are consistent with an epithelial carcinoma, and like human ovarian carcinoma, it expresses her-2/neu, sex hormone receptors, and characteristic cytokines. FNAR also displays a similar gene expression pattern to the human disease. Consistent with the endometrioid subtype, FNAR cells show cell-surface expression of CA125 and nuclear expression of β-catenin.

The FNAR model may address many of the limitation of current model systems for ovarian carcinoma. Rats transplanted with FNAR consistently become moribund by 5-6 months, avoiding the low frequency and long latency of spontaneous animal models. Xenografts of primary human tumors in immunodeficient mice are perhaps the most attractive current model [16-19]. Although spontaneous human cancers can be studied and used to test treatments in these mice, the study of immunotherapeutic approaches is problematic. Conversely, FNAR develops in immunocompetent rats, allowing the study of immunotherapeutic approaches. The expression of all three sex hormone receptors and her-2/neu also allows for manipulations of these pathways using this model. However, the application of this model to the treatment of human disease remains to be established.

## Competing interests

The authors declare that they have no competing interests.

## Authors' contributions

AS, RJ, and AH designed research. AS, CT, BR, and JB performed research. RG, DA, and CT gave assistance in analyzing model. AS, RJ, AH, and BR wrote manuscript. All authors have read and approved the final manuscript.
